# Lemon seed allergy: a case presentation

**DOI:** 10.1186/s13223-020-00429-x

**Published:** 2020-04-29

**Authors:** O. Stephanie Kayode, Nuevalynne Prado, David J. Thursfield, Stephen J. Till, Leonard Q. C. Siew

**Affiliations:** 1 Department of Adult Allergy, Guy’s and St Thomas’ NHS Foundation Trust, London, SE1 9RT UK; 2 Peter Gorer Department of Immunobiology, School of Immunology and Microbial Sciences, King’s College London, London, UK

**Keywords:** Adult, Citrus, Seed, Anaphylaxis, Immunoglobulin E, Food hypersensitivity, Tree Nut, Case report, Hybridisation

## Abstract

**Background:**

We report a case of IgE-mediated hypersensitivity to lemon seed. We demonstrate for the first time a pattern of cross-sensitisation between seeds of citrus hybrid species from similar ancestral species origins.

**Case report:**

Described is a case of a 26-year-old female with recurrent anaphylaxis on exposure to lemon seed with sensitisation shown on prick to prick testing. Prick to prick testing was also performed to a variety of citrus fruit seeds and edible foods from additional notable families of the Sapindale order.

**Conclusion:**

In cases of unexplained or recurrent anaphylaxis in adult patients, citrus seed allergy should be considered.

## Background

Citrus fruits belong to the Citrus genus of the Rutaceae family and Sapindale order. Mature citrus fruits consist of an outer peel (epicarp), thick skin (mesocarp) and juice filled flesh (endocarp). Most also contain seeds. They are consumed in a variety of foods and drinks worldwide. Recognised citrus allergens include: germ-like protein, profillin and lipid transfer protein [1, 2]. Allergens identified within the citrus fruit seeds are citrin: a globulin seed storage protein, and albumin seed storage proteins [3].

We present the first adult case of lemon seed allergy with a demonstrable pattern of cross-sensitisation within the citrus fruit species.

## Case presentation

A 26-year-old woman was referred to the Allergy Clinic for assessment of recurrent anaphylaxis. She presented with nasal congestion, wheeze, throat tightness, generalised urticaria and nausea and vomiting immediately following salad consumption. Her symptoms resolved within 1 h with oral antihistamine and salbutamol inhalation. She did not seek emergency care. She had two further episodes on salad consumption. A common ingredient in these salads was freshly hand squeezed lemon. She tolerated lemon juice and orange juice in drinks without symptoms. Her medical history included pistachio and cashew nut allergy as well as asthma.

Skin prick testing (SPT) performed to commercial extracts were positive to grass pollen (3 mm wheal diameter, Allergy Therapeutics). Tree pollens (Allergy Therapeutics) in particular Birch pollen (Allergopharma GmbH & Co, Germany) and peach (surrogate marker for lipid transfer protein, ALK-Abello, Madrid, Spain) were negative.

Prick to prick testing (PPT) was positive to lemon seed (25 mm wheal diameter) and negative to lemon peel and flesh, Table 1. The patient was diagnosed to have new IgE-mediated hypersensitivity to lemon seed. PPT was also performed to other citrus fruits and edible foods from additional notable families of the Sapindale order, Table 1.

**Table 1:** Prick to prick testing wheal diameter results in mm

	Seed	Flesh	Peel
Rutaceae family			
Lemon	*25*	0	0
Limequat	*17*	2	0
Orange	*10*	0	0
Grapefruit	0	0	0
Clementine	0	0	0
Bergamot	0^a^	0	3
Pomelo	0	0	0
Kumquat	0	0	0
Anacardiaceae family			
Mango	*3*	0	0
Cashew	*17*	N/A	N/A
Pistachio	*15*	N/A	N/A
Sapindaceae family			
Lychee	0	0	0

^a^Of note, underdeveloped seeds were present within the bergamot citrus fruit

All skin prick and prick to prick tests were validated with positive histamine and negative normal saline controls (Allergy Therapeutics). At the time of skin testing, seedless limes were the only commercially available lime species in the region. We were therefore unable to perform PPT to lime species.

## Discussion and conclusion

**Fig. 1:**
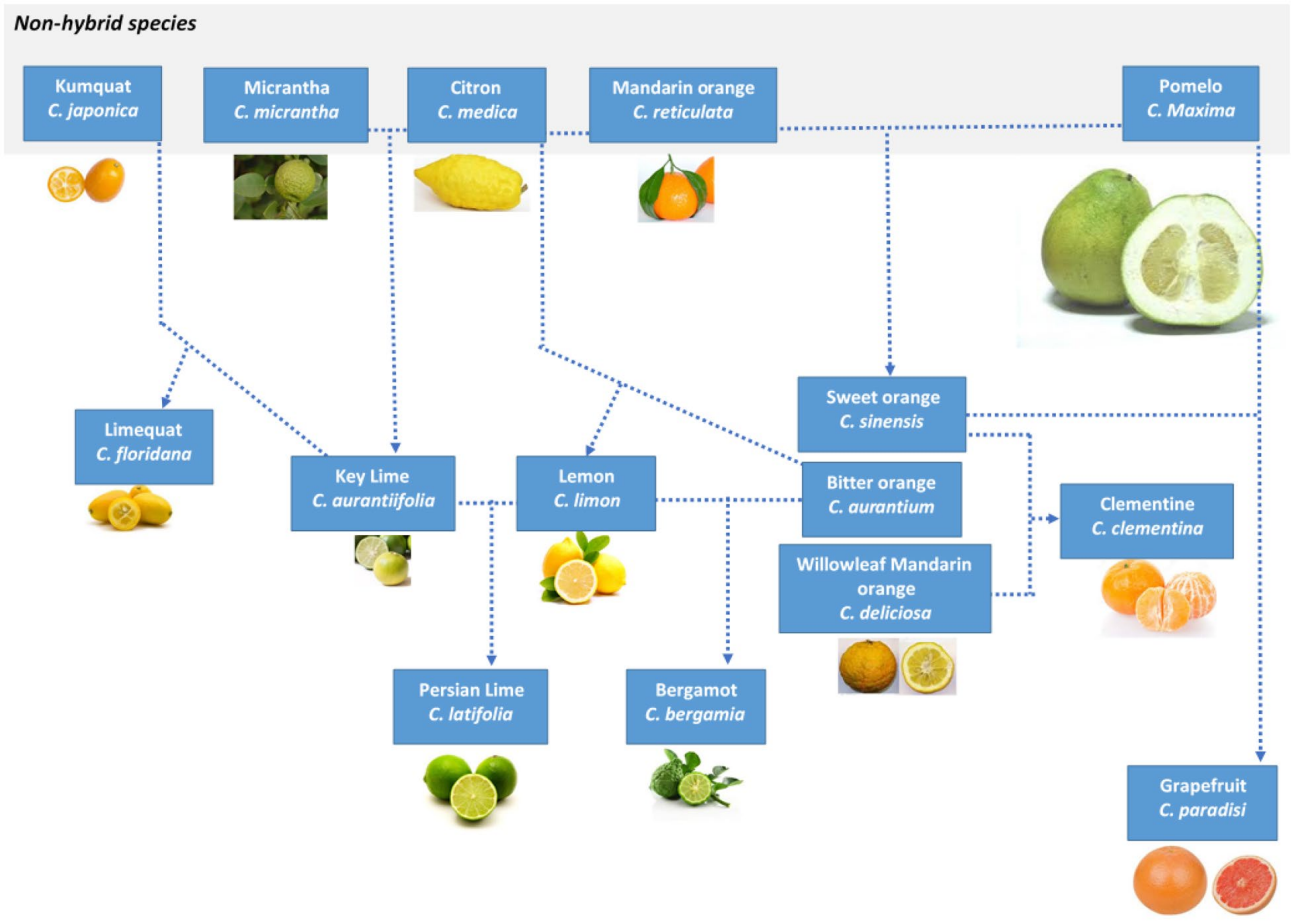
Hybridisation of various citrus species from their ancestral species

Citrus fruits are produced from flowering citrus plants, which are native to South Asia and are cultivated throughout the world [4]. Genomic and phylogenetic analysis reveals most citrus species are derived from core ancestral citrus species: citron (*C. medica*), pomelo (*C. maxima*), mandarin orange (*C. reticulata*), kumquat (*C. japonica*) and micrantha (*C. micrantha*) [4]. Ancestral citrus species have undergone inter-breeding and hybridisation to create a diversity of hybrid species, Fig. 1. Citrus hybrid species with shared ancestral origins display closer genetic similarities [4].

Immediate hypersensitivity reactions to the flesh of citrus fruit vary in severity grade from mild oral allergy syndrome to anaphylaxis. These reactions can be augmented by exercise and NSAID co-factors [5]. Delayed hypersensitivity reactions have also been reported to both citrus flesh and peel namely allergic contact dermatitis [6].

Citrus seeds are mainly composed of oils, proteins and fibre. Citrus seed allergy is uncommon and case reports are limited in number. Cross-reactivity has been demonstrated between seeds of various citrus fruits including orange, mandarin and lemon [7, 8] as well as with peanut [8]. Cross-reactivity between citrus fruit seeds and other tree nuts have only been reported in children [9].

Presented is a case of recurrent anaphylaxis due to lemon seed allergy. Our patient demonstrated allergic co-morbidities between seeds of two families of the Sapindale order (Rutaceae and Anacardiaceae families). While it is possible seeds of the Rutaceae and Anacardiaceae family share cross-reactive antigenic seed storage protein epitopes as demonstrated in children [9], confirmatory immunoblotting laboratory testing in adults is required before cross-reactivity between both families can be proposed in adult patients. Our patient did not demonstrate cross-sensitisation between seeds of the Rutaceae and Sapindaceae family.

An emerging pattern of cross-sensitisation between seeds of various citrus fruit species was also observed. Cross-sensitisation occurred between hybrid species derived from similar ancestral species: the citron (*C. medica*) and mandarin orange (*C. reticulata*) ancestral species. Based on hybridisation knowledge we had expected the patient to have a positive result to bergamot seed. However, seeds within the sourced bergamot fruit were underdeveloped and therefore potentially lacked sufficient quantities of seed storage protein allergens to elicit a positive prick to prick test result.

In conclusion, citrus seed hypersensitivity although rare, should be considered in cases of unexplained or recurrent anaphylaxis in adult patients. Without this high index of suspicion, the history of accidental seed consumption can be easily overlooked.

## Data Availability

All data generated or analysed during this study are included in this published article.

## Abbreviations

SPT: Skin prick testing
PPT: Prick to prick testing
